# NT-proBNP levels, atherosclerosis and vascular function in asymptomatic type 2 diabetic patients with microalbuminuria: peripheral reactive hyperaemia index but not NT-proBNP is an independent predictor of coronary atherosclerosis

**DOI:** 10.1186/1475-2840-10-71

**Published:** 2011-08-03

**Authors:** Henrik Reinhard, Niels Wiinberg, Peter R Hansen, Andreas Kjær, Claus L Petersen, Kaj Winther, Hans-Henrik Parving, Peter Rossing, Peter K Jacobsen

**Affiliations:** 1 Steno Diabetes Center, Gentofte, Denmark; 2 Department of Clinical Physiology and Nuclear Medicine, Frederiksberg University Hospital Frederiksberg, Denmark; 3 Department of Cardiology, Gentofte University Hospital, Gentofte, Denmark; 4 Department of Clinical Physiology, Nuclear Medicine & PET and Cluster for Molecular Imaging, University of Copenhagen, Copenhagen, Denmark; 5 Department of Clinical Biochemistry, Frederiksberg University Hospital, Frederiksberg, Denmark; 6 Department of Medical Endocrinology, University Hospital of Copenhagen, Copenhagen, Denmark; 7 Faculty of Health Science, Aarhus University, Aarhus, Denmark; 8 The Heart Centre, University Hospital of Copenhagen, Copenhagen, Denmark

## Abstract

Intensive multifactorial treatment aimed at cardiovascular (CV) risk factor reduction in type 2 diabetic patients with microalbuminuria can diminish fatal and non-fatal CV. Plasma N-terminal (NT)-proBNP predicts CV mortality in diabetic patients but the utility of P-NT-proBNP in screening for atherosclerosis is unclear. We examined the interrelationship between P-NT-proBNP, presence of atherosclerosis and/or vascular dysfunction in the coronary, carotid and peripheral arteries in asymptomatic type 2 diabetic patients with microalbuminuria that received intensive multifactorial treatment.

**Methods and Results:**

P-NT-proBNP was measured in 200 asymptomatic type 2 patients without known cardiac disease that received intensive multifactorial treatment for CV risk reduction. Patients were examined for coronary, carotid and peripheral atherosclerosis, as defined by coronary calcium score ≥400, carotid intima-media thickness (CIMT) > 0.90 mm, ankle-brachial index < 0.90, and/or toe-brachial index < 0.64, respectively. Carotid artery compliance was also determined and the reactive hyperaemia index (RHI) measured by peripheral artery tonometry was used as a surrogate for endothelial function.

P-NT-proBNP was associated with atherosclerosis in the unadjusted analysis, but not after adjustment for conventional risk factors. P-NT-proBNP was not associated with vascular dysfunction. The prevalence of atherosclerosis in the coronary, carotid and peripheral arteries was 35%, 10% and 21% of all patients, respectively. In total 49% had atherosclerosis in one territory and 15.6% and 1.0% in two and three territories. Low RHI was an independent predictor of coronary atherosclerosis (odds ratio [CI], 2.60 [1.15-5.88] and systolic blood pressure was the only independent determinant of CIMT (0.02 mm increase in CIMT per 10 mmHg increase in systolic blood pressure [p = 0.003]).

**Conclusions:**

Half of asymptomatic patients with type 2 diabetes mellitus and microalbuminuria had significant atherosclerosis in at least one vascular territory despite receiving intensive multifactorial treatment for CV risk reduction. Coronary atherosclerosis was most prevalent, whereas carotid disease was more rarely observed. RHI but not plasma NT-proBNP was predictive of coronary atherosclerosis.

## Introduction

Atherosclerosis is the most important determinant of the excessive morbidity and mortality in type 2 diabetic patients, especially in patients with albuminuria. Medical treatment aimed at reduction of established cardiovascular (CV) risk factors is effective in reducing the increased CV morbidity and mortality in diabetic patients with microalbuminuria [1]. By enabling early and aggressive preventive interventions, screening for subclinical atherosclerotic disease may therefore be of value in high risk diabetic patients.

Elevated plasma brain natriuretic peptide (P-BNP) and N-terminal-proBNP (P-NT-proBNP) levels, released in response to cardiomyocyte stress, are established risk factors in patients with heart failure [2]. In addition, minor increases in P-NT-proBNP below levels seen in heart failure, have been associated with poor outcome in other populations [3]. We have previously identified P-NT-proBNP as a powerful predictor of CV mortality in type 2 diabetic patients, independent of albuminuria [4]. The causes of the poor prognosis associated with elevated P-NT-proBNP are unclear but elevated P-NT-proBNP may correlate with the severity of coronary atherosclerosis [5]. Furthermore, whether P-NT-proBNP is associated with subclinical atherosclerotic manifestations or vascular dysfunction in the carotid or peripheral arteries is not known in detail. Finally, if the presence of atherosclerosis and/or dysfunction in different territories are correlated in type 2 diabetic patients that receive intensive multifactorial treatment, aimed on CV prevention, is not known [6]. Accordingly, we examined the interrelationship between P-NT-proBNP, presence of atherosclerosis and/or vascular dysfunction in the coronary, carotid and peripheral arteries, respectively, in asymptomatic type 2 diabetic patients with microalbuminuria that received intensive multifactorial treatment.

## Methods

### Patient cohort and clinical measurements

In a cross-sectional study at Steno Diabetes Center, we identified from January 2007 to February 2008, a consecutive cohort of 200 asymptomatic type 2 diabetic patients with microlbuminuria without prior known CV disease and with normal plasma creatinine to allow for examinations with x-ray contrast media. Diabetes was diagnosed by the WHO criteria and all patients received intensive multifactorial intervention aimed at optimal glycaemic, lipid, and blood pressure control, as well as antiplatelet therapy and lifestyle modification according to the Steno-2 study [1]. Noteworthy, an audit compared the levels of treatment targets before and after the Steno-2 study (2002 and 2009) and demonstrated that the Steno-2 study results of lowering haemoglobinA1c, lipids and blood pressure were successfully implemented into clinical practise at Steno Diabetes Center [7].

The clinical characteristics and P-NT-proBNP measurements have previously been described, including our NT-proBNP cut-off value of 45.2 ng/l, which represented the median P-NT-proBNP in the first 50 patients examined in the study [8]. Tests for autonomic neuropathy, heart rate variability assessed by the expiration-inspiration variation of the heart rate, and orthostatic blood pressure measurements were performed. Heart rate variability ≤ 10 bpm and an orthostatic blood pressure fall > 30 mmHg were considered abnormal. Somatic nerve function (vibratory perception threshold) was evaluated by biothesiometry. Smoking was defined as persons smoking one or more cigarettes/cigars/pipes a day, all others were classified as non-smokers.

### Investigations of the coronary, carotid, and peripheral arteries

Coronary calcium scanning was performed during a single breath hold using a 16 multidetector-row CT scanner with 3 mm slice thickness (Philips Precedence MX 8000 IDT 16 slice, Philips Medical Systems, Best, The Netherlands). Quantification of Agatston coronary calcium score (CCS) was done as previously described [9]. CCS is a continuous measurement of the coronary arteries atherosclerotic burden and CCS is strongly correlated with histopathologic coronary artery disease[10 11]. Furthermore, CCS is a powerful predictor of CV morbidity and mortality in asymptomatic diabetic and non-diabetic populations [12]. Of note, in diabetic patients with proteinuria a threshold (CCS≥400) for clinical significant disease exists, and accordingly, coronary atherosclerosis was defined as CCS≥400 [13]. Patients with NT-proBNP ≥ 45.2 ng/l and/or CCS ≥ 400 were further examined with myocardial perfusion imaging (MPI), CT angiography (CTA) and/or coronary angiography (CAG), but the correlations between P-NT-proBNP, CTA, MPI and CAG have previously been reported [8]. Therefore in the current study, the results of the latter investigations were not used for our definition of coronary atherosclerosis (6). A common carotid artery wall segment of 5-10 mm in length was imaged in a longitudinal view, located approximately 20 mm proximal to the bifurcation (Siemens Acuson Cypress ultrasound scanner with a linear probe 7-10 MHz 7L3). Carotid intima-media thickness (CIMT) was measured at the posterior arterial wall. Bilateral carotid ultrasound examination was performed and the mean CIMT of both arteries were used. Carotid artery atherosclerosis was defined as CIMT > 0.90 mm [14]. All images were saved as 4- to 6-second long dynamic scans and arterial compliance was measured with dedicated vascular research tools (Medical Imaging Applications [MIA] version 5.0; LLC, Coralville, IA, USA). Carotid vascular function was assessed by regional vascular compliance of the carotid arteries, using B-mode scans and calculated as previously described, i.e., carotid compliance = (carotid diameter_max_^2 ^- carotid diameter_min_^2^)/pulse pressure [14]. Carotid compliance was expressed as arterial distensibility adjusted for vessel size and sex.

Systolic blood pressures in the ankle and big toe were measured on both legs by the strain gauge technique and the lowest pressures were used for calculation of the ankle-brachial index (ABI) and toe-brachial index (TBI) index, respectively [15]. Peripheral artery disease was defined as ABI < 90% and/or TBI < 64% [16].

Finally, peripheral microvascular endothelial function and arterial stiffness were measured with a plethysmographic device (EndoPAT2000, Itamar Medical Ltd, Caesarea, Israel). This test was presented to the patients as a scientifically important but optional test since it is not yet applicable in a clinical setting. As previously described, a pulse wave amplitude was obtained with pneumatic probes in both test and control index fingers during 10 minutes rest in the supine position and again after interrupting the brachial arterial blood flow by inflation a blood pressure cuff (>200 mmHg) for 5 minutes in the test finger [17]. The reactive hyperaemia index (RHI) and augmentation index (AI) were obtained with computerized automated algorithm analyses. RHI was the ratio of postdeflation to baseline pulse amplitude in test finger divided by the same ratio in control finger. RHI is dependent on endothelium-derived nitric oxide availability and is positively correlated with both the flow-mediated vasodilation measured in the brachial artery and the endothelium-dependent coronary flow reserve[18 19]. AI is defined as the difference between the first and the second peak of the radial arterial waveform by return of reflected pressure waves [20]. Peripheral artery AI is an index of arterial stiffness, i.e., when arteries are stiffer, wave reflections return earlier, and is positively correlated with central arterial stiffness [21].

The study was approved by the local ethics committee and all patients gave written informed consent.

### Statistical analysis

Our primary objective was to describe if patients with elevated NT-proBNP (and therefore poor prognosis) have subclinical atherosclerosis and/or vascular dysfunction. Accordingly, we investigated if patients with P-NT-proBNP levels above our previous defined cut-off value (45.2 ng/l) had more atherosclerosis and/or dysfunction than patients with P-NT-proBNP below this value [22]. Furthermore, we examined the associations between atherosclerosis or vascular dysfunction and CV risk factors including P-NT-proBNP as a continuous variable in univariate linear regression analyses and multivariate logistic regression models with determination of unadjusted odds ratios (ORs) and ORs adjusted for variables associated with atherosclerosis/vascular dysfunction in the univariate analysis. In addition, the predictive accuracy for presence of atherosclerosis of the covariate-adjusted models with and without inclusion of P-NT-proBNP were compared by generating receiver operating characteristic (ROC) curves and the areas under the ROC curves (AUCs) were calculated. In the above analyses, patients were divided into groups of patients with or without atherosclerosis as determined by CCS, CIMT, ABI and/or TBI, and subgroups including patients with coronary, peripheral or carotid atherosclerosis alone, and in patients with atherosclerosis in two or three vascular territories, respectively. Comparisons between groups were performed by unpaired Student's t-test or analysis of variance (ANOVA). The Pearson Chi^2^-test was used to compare non-continuous variables. Secondarily, we evaluated if cut-offs (quartile) values of the functional vascular/atherosclerotic tests in multivariate logistic regression were able to identify atherosclerosis in different vascular territories but in particular coronary atherosclerosis. Data were expressed as means (SD), except for non-normally distributed variables, which were log10-transformed before analysis and are given as medians (interquartile range). A P-value less than 0.05 was considered as statistically significant. All data were analyzed by using statistical package for social sciences (SPSS) version 14 for Windows.

## Results

### Patient characteristics

The clinical characteristics of patients are summarized in Table 1. Of note, the medical treatment for CV disease prevention included statins (94% of patients), aspirin (90%), and renin angiotensin aldosterone system (RAAS)-blocking agents (98%).

**Table 1:** Clinical characteristics in 200 type 2 diabetic patients and in 173 patients with or without atherosclerosis, including coronary calcium score (CCS) ≥400, ankle-brachial index (ABI) < 0.90 and/or toe-brachial index (TBI) < 0.64 and/or carotis intima-media thickness (CIMT) > 0.90 mm, respectively

	All patients (n = 200)	Patients with atherosclerosis (n = 85) ^	Patients without atherosclerosis (n = 88) ^	p-values
Sex no. (male%)	152 (76)	73 (86)	59 (67)	0.004
Age (years)	59 (9)	62 (5)	55 (9)	<0.001
Duration of diabetes (years)	13 (7)	14 (7)	11 (7)	0.001
BMI (kg/m^2^)	32.6 (5.8)	31.5 (5.3)	33.5 (6.2)	0.029
HbA_1c _(%)	7.9 (1.3)	7.8 (1.2)	7.9 (1.5)	0.62
Urinary albumin excretion rate (mg/24h)*	103 (39 - 230)	98 (33-293)	87 (44-191)	0.85
P-creatinine (μmol/l)	76 (18)	79 (18)	74 (18)	0.13
Systolic blood pressure (mmHg)	130 (17)	132 (18)	128 (16)	0.16
Total cholesterol (mmol/l)	3.9 (0.9)	3.9 (1.0)	4.0 (0.9)	0.58
Heart rate variation during deep breathing (bpm)*	7 (4.5-11.5)	6 (4-9)	8 (5-13)	0.013
Current smoker no. (%)	59 (30)	31 (36)	19 (22)	0.031
Vibratory perception threshold mV - mean of both sides	33 (15)	36 (14)	30 (15)	0.004
Retinopathy no. (%)	120 (60)	57 (67)	41 (48)	0.010
Abnormal heart rate variation during deep breathing no. (%)	118 (59)	61 (72)	51 (58)	0.015
Ortostatic hypotension no. (%)	15 (7.5)	8 (9)	6 (7)	0.055
Oral antidiabetic medication no. (%)	170 (85)	70 (82)	76 (86)	0.47
Insulin treatment no. (%)	124 (62)	56 (67)	51 (58)	0.28
RAAS blockade no. (%)	196 (98)	82 (96)	87 (99)	0.30
Statin therapy no. (%)	189 (95)	78 (92)	85 (97)	0.20
Aspirin therapy no. (%)	183 (92)	79 (93)	82 (93)	0.95
Beta-blocker therapy no. (%)	27 (14)	14 (16)	11 (13)	0.46
Calcium channel blockers no. (%)	80 (40)	36 (41)	30 (35)	0.26
Use of diuretics no. (%)	128 (64)	58 (66)	53 (62)	0.27
NT-proBNP (ng/l) *	48.7 (18.6-95.0)	44.2 (24.5-108.5)	31.9 (12.7-95.0)	0.021
NT-proBNP > 45.2 (ng/l) no. (%)	104 (52)	49 (58)	40 (45)	0.11
NT-proBNP ≤ 45.2 (ng/l) no. (%)	96 (48)	36 (42)	48 (55)	0.11

Data are expressed as means (SD) or medians (interquartile range) *, nr = not relevant, ^a^Our plasma NT-proBNP (45.2 ng/l) cut-off has previously been described [8],. ^ In five, seven and 17 patients CCS, peripheral systolic blood pressure and CIMT were not measured respectively, and we only included patients with structural tests in all three territories (173/200).

### Plasma NT-proBNP and atherosclerosis

Patients with P-NT-proBNP above our cut-off value (45.2 ng/l) did not have more overall atherosclerosis or more vascular dysfunction than patients with NT-proBNP levels below this value (p > 0.05). We did, however, demonstrate that coronary atherosclerosis was more present in patients with P-NT-proBNP above cut-off value (42 vs. 26 patients p = 0.041). Furthermore, P-NT-proBNP levels were higher in patients with atherosclerosis in one vascular territory (median [interquartile range]; 47.5 [23.1-83.8] ng/l) and in patients with atherosclerosis in two or three vascular territories (90.6 [25.3-227.5 ng/l]) compared to patients without atherosclerosis (32.6 (12.7-95.0) ng/l, p = 0.005). However, after adjustments for age and gender these associations were no longer significant (p > 0.05). Finally, adding P-NT-proBNP to the ROC curve for prediction of atherosclerosis did not increase the AUC (78.4% vs. 78.4%, not shown).

### Atherosclerosis in different vascular territories: Prevalences and risk factors

Although a few patients were not examined with all investigative modalities due to technical difficulties, CCS was obtained in 98% (195/200) of patients and ultrasonography of the common carotid artery was performed in 92% (183/200) of patients, including a bilateral examination in 77% (147/200) of patients. Peripheral systolic blood pressure including a toe pressure was measured in 97% (193/200) and peripheral artery tonometry was performed in 83.5% (167/200) of patients. We experienced no technical problems with the latter test but some patients declined this procedure.

In total, 173 patients had examinations of all three vascular territories and among these patients 49.1% (85/173) had abnormalities indicative of atherosclerosis as defined in the current study, in at least one territory. Of the 27 patients excluded in this analysis, all patients but one patient had 2 vascular investigations performed, including 10 and 16 patients with or without signs of atherosclerosis, respectively. The clinical characteristics and results of the vascular measurements in all patients (n = 200) and in patients that underwent all three investigations (n = 173) are summarized in Table 2. In a multivariate logistic regression model, including risk factors associated with atherosclerosis, i.e., age, sex, diabetes duration, smoking, retinopathy, body mass index (BMI), heart rate variability, vibration threshold and NT-proBNP, age (OR [CI] 1.14 [1.06-1.22]), sex (male, 4.31 [1.60-11.88]) and diabetes duration (1.07 [1.00-1.14]) remained independent predictors of atherosclerosis.

**Table 2:** Atherosclerosis measurements in 200 type 2 diabetic patients and in 173 patients with or without atherosclerosis, including coronary calcium score (CCS) ≥400, ankle-brachial index (ABI) < 0.90 and/or toe-brachial index (TBI) < 0.64 and/or carotis intima-media thickness (CIMT) > 0.90 mm, respectively

	All patients (n = 200)	Patients with atherosclerosis (n = 85) ^	Patients without atherosclerosis (n = 88) ^	p-values
Coronary Calcium Score*	183 (6-604)	552 (256-1369)	12 (0-115)	nr
Carotid intima-media thickness (mm)	0.73 (0.15)	0.79 (0.17)	0.68 (0.10)	nr
Carotid function^a ^(l/mmHg)	0.0026 (0.001)	0.0024 (0.001)	0.0027 (0.001)	0.073
Toe-brachial index (%)	121 (34)	75 (23)	100 (17)	nr
Ankle-brachial index (%)	108 (17)	98 (19)	114 (11)	nr
Pulse pressure wave augmentation ^b^*	5 (-2-12)	8 (0-14)	3 (-5-10?)	0.076
Reactive hyperemia index ^c^	1.70 (0.44)	1.64 (0.42)	1.75 (0.47)	0.14
Reactive hyperemia index ≤1.43 ^^^^	45 (27)	26 (33)	14 (20)	0.007
Reactive hyperemia index > 1.43 ^^^^	122 (73)	43 (55)	64 (93)	0.007
CAD^d ^no. (%)	70 (35)	44 (52)	16 (18)	<0.001

Data are expressed as means (SD) or medians (interquartile range) *, nr = not relevant, ^a^carotid distensibility measured with a ultrasound scanner, peripheral microvascular endothelial function measured as ^b^radial augmentation index or ^c^reactive hyperemia index, both measured with a plethysmographic device. ^d ^Significant coronary artery disease (CAD) as defined by myocardial perfusion imaging and coronary angiography and previous described [8]. ^ In five, seven and 17 patients CCS, peripheral systolic blood pressure and CIMT were not measured respectively, and we only included patients with structural tests in all three territories (173/200). ^^ Reactive hyperemia index was performed in 167/200 patients and 147 of the 167 patients also had all three structural tests performed.

Coronary atherosclerosis as defined by CCS≥400 was found in 34.9% (68/195) of patients and these patients were older (mean age 63 vs. 57 years, p = 0.0001), more often males (85% vs. 71% p = 0.025), and had longer diabetes duration (15 vs. 12 years, p = 0.003), more retinopathy (p = 0.045), higher vibratory perception threshold (38 vs. 31 mV, p = 0.002) and higher P-NT-proBNP (median values 64.4 vs. 42.4 ng/l, p = 0.006) compared with patients with CCS < 400. In a multivariate logistic regression analysis including the above variables, only age remained independently associated with coronary atherosclerosis.

Carotid atherosclerosis as defined by CIMT > 0.90 mm was found in 10.4% (19/183) of patients and here only age (68 vs. 58 years, p = 0.015) was significantly increased compared to patients without disease in this vascular territory. However in a linear regression analysis with CIMT as the dependent variable, weak associations were found between CIMT and patient age (R = 0.30, p = 0.0001), systolic blood pressure (R = 0.22, p = 0.003), vibratory perception threshold (R = 0.18, p = 0.016), heart rate variability (-R = 0.15, p = 0.053), presence of retinopathy (R = 0.17, p = 0.019, toe-brachial index (R=-0.22, p = 0.002), P-NT-proBNP (R = 0.19, p = 0.009) and CCS (R = 0.19, p = 0.009). Including these variables in multivariable linear regression analysis, only systolic blood pressure was significantly associated with CIMT, with a 0.015 mm increase in CIMT for every 10 mmHg increase in systolic blood pressure (p = 0.027). Furthermore, a borderline non-linear relationship between CIMT and diabetes duration was found, i.e., when dividing diabetes duration in to quartiles (<7,7-12, 12-18, and > 18 years), means of CIMT were 0.697, 0.743, 0.773, and 0.709 mm (ANOVA, p = 0.054), respectively.

Peripheral artery disease as defined by ABI < 90% and/or TBI < 64% was demonstrated in 20.7% (40/193) of patients and in addition to the associations also seen between certain risk factors (age, sex, duration, vibratory perception threshold, and P-NT-proBNP) and coronary atherosclerosis, these patients also had higher P-creatinine (80.3 vs. 74.2 μmol/l, p = 0.052), lower heart rate variability (median 5 vs. 8 bpm, p = 0.003) and lower BMI (30.3 vs. 32,7 kg/m2, p = 0.02) compared to patients without peripheral atherosclerosis. In a multivariate logistic regression model, including above-mentioned risk factors, only BMI was independently associated with peripheral artery disease, (p = 0.045).

### Interrelations between atherosclerosis in different vascular territories

**Figure 1:**
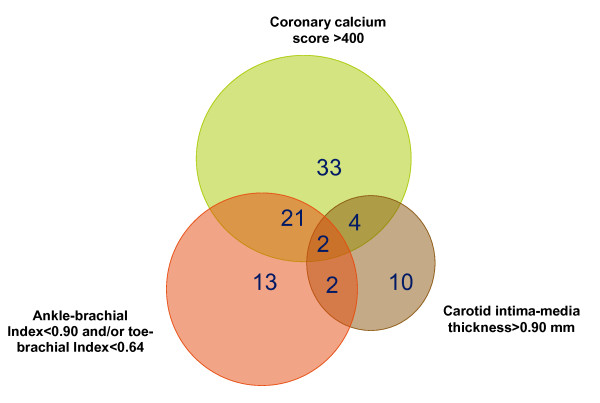
**Venn diagram showing the relationship between cardiovascular disease manifestations in different territories in 85 type 2 diabetic patients with microalbuminuria**.

Among 56 patients with atherosclerosis in only one vascular territory (Figure 1), no differences in risk factors were demonstrated when comparing patients with coronary, carotid, and peripheral atherosclerosis (ANOVA, p > 0.05). As shown in Figure 1, only 15.6% (29/173) of patients had atherosclerosis in at least two territories, including 2 patients with atherosclerosis in all three territories. Patients with atherosclerosis in two or three territories only had higher vibratory perception threshold (42 vs. 33 mV, p = 0.013) compared to patients with atherosclerosis in only one territory, while the other risk factors were comparable between groups. The level of microalbuminuria was not associated with atherosclerosis in any vascular territory.

Peripheral artery disease was not directly related with atherosclerosis in the coronary territory (Figure 1). Although atherosclerosis was confined to one vascular territory in the majority of cases, patients with atherosclerosis in one territory had significantly higher risk of having disease in another territory, i.e., unadjusted and adjusted (for age, sex, diabetes duration, retinopathy, vibration threshold, and P-NT-proBNP) ORs for coronary atherosclerosis were 3.52 (1.89-8.11) and 2.62 (1. 20-5.974) in patients with peripheral artery disease, and unadjusted and adjusted (for age, sex, diabetes duration, P-creatinine, heart rate variability, BMI, vibration threshold, and P-NT-proBNP) ORs of peripheral artery disease were 3.92 (1.89-8.11) and 3.38 (1.46-7.81) in patients with coronary atherosclerosis.

### Peripheral vascular function and carotid distensibility

RHI and AI measured by peripheral artery tonometry and data on carotid distensibility assessed by ultrasound examination are shown in Table 1. AI and carotid distensibility did not differentiate between atherosclerosis vascular territories and extent of the disease (1, 2 or 3 territories), but with use of a cut-off value, RHI was found to contain important differential information. When RHI was divided into quartiles and patients in the lower quartile (RHI ≤1.43 [low RHI], n = 45) were compared with patients in the other quartiles (normal RHI, n = 122) in multivariate logistic regression analyses with coronary atherosclerosis or peripheral artery disease, or the number of diseased vascular territories as dependent variables, respectively, and adjustments were made for the previously described risk factors, low RHI was independently associated with coronary artery atherosclerosis (OR 2.60 [1.15-5.88]), peripheral artery disease (OR 3.60 [1.45-8.95]), and atherosclerosis in any vascular territory ( OR 3.28 [1.30-8.25]). It is noteworthy, that when the presence of peripheral artery disease was included in the predictive model, low RHI remained independently predictive of coronary atherosclerosis (p = 0.04). The prevalences of low and normal RHI in patients with or without atherosclerosis are shown in Table 1. Among patients with atherosclerosis in two or three vascular territories, 48% had low RHI compared to 33% in patients with atherosclerosis in one territory (p = 0.23). In addition, 72% of the patients with normal RHI were free of coronary atherosclerosis (p = 0.022) and in patients with normal RHI or age below 57 (the 1^st ^age tertile) years (n = 40), 95% were without coronary atherosclerosis (p < 0.001). In contrast, age below 57 years alone excluded coronary atherosclerosis in 84% of patients (p < 0.001).

## Discussion

### Principal findings

Our primary objective was to describe if type 2 diabetic patients with microalbuminuria and with elevated NT-proBNP (and therefore poor prognosis) have subclinical atherosclerosis and/or vascular dysfunction. NT-proBNP levels were not related with vascular dysfunction, and although NT-proBNP levels were significantly associated with the presence and extent of atherosclerosis, we demonstrated no independent associations after adjustments for age and sex. Secondarily, we determined the prevalence of atherosclerosis in 200 asymptomatic type 2 diabetic patients with microalbuminuria but without known CV disease and with normal plasma creatinine. Among these patients that received intensive multifactorial treatment aimed at CV risk reduction, almost half of subjects had atherosclerosis in at least one vascular territory, and 15% and 1% had atherosclerosis in two and three territories including coronary, carotid and peripheral arteries, respectively. Furthermore, we found that carotid atherosclerosis was rarely observed (10%) and was independently associated with the systolic blood pressure, whereas coronary atherosclerosis was more prevalent (35%) and was associated with low RHI measured by peripheral tonometry.

### Increased NT-proBNP and atheroslcerosis

We have previously demonstrated a strong and independent risk for CV mortality with elevated P-NT-proBNP in type 2 diabetic patients [4]. In the present study, however, no independent relationship was found between P-NT-proBNP and atherosclerosis. Several factors may contribute to this finding. First, patients in this study were asymptomatic and had normal P-creatinine wheareas subjects in our earlier study were all type 2 diabetic patients (e.g., including cases with CV disease and elevated P-creatinine) followed in an out-patient clinic (4). Second, the increasingly intensive treatment used today in our patients is likely to have influenced the results compared to the earlier study, although in the Steno 2 multifactorial intervention study, P-NT-proBNP remained a strong independent predictor of CV mortality and here NT-proBNP levels also increased over time in the intervention group [23].Third, other factors than atherosclerosis per se, e.g., cardiac autonomic neuropathy or echo abnormalities are likely to influence CV mortality. Along this line, it has been demonstrated that half of asymptomatic type 2 diabetes patients have left ventricular diastolic dysfunction and that BNP is associated with presence of diastolic dysfunction [24]. Actually, BNP is a useful tool for screening preclinical moderate-severe diastolic dysfunction, which could be irrespective of age and obesity[25 26]. In addition measuring NT-proBNP before non cardiac surgery in clinical practice is useful to risk stratify patients [27]. Finally, BNP is also a sensitive and specific biomarker for the detection of systolic dysfunction in the general population [28].

### Multifactorial treatment and subclinical atherosclerosis

Intensive individual and multifactorial interventions including polypharmacologic therapy according to international guidelines is the current standard of care in our institution and is known to reduce CV morbidity and mortality [1]. Noteworthy, an audit compared the levels of treatment targets before and after the Steno-2 study (2002 and 2009) and demonstrated that the Steno-2 study results of lowering haemoglobinA1c, lipids and blood pressure were successfully implemented into clinical practise at Steno Diabetes Center [7]. Almost all patients in the current study received statins, aspirin and RAAS blockading agents, and 33%, 28%, and 17% received 2, 3, and ≥4 antihypertensive drugs on top of RAAS blockade. Indeed, this intensive treatment yielded mean total cholesterol levels of 3.9 mM, arterial blood pressures of 130/75 mmHg, and haemoglobinA1c levels of 7.9%, respectively, which may explain why most of the conventional CV risk factors as well as albuminuria were not associated with atherosclerosis in our study.

At birth CIMT is 0.5 mm and with time CIMT increases which is accelerated in the presence of CV risk factors. A novel finding of the current study was the low prevalence of carotid artery atherosclerosis in our patients, i.e., CIMT > 0.9 mm in 10.4% and CIMT > 1.0 mm in 4.6% of patients (not shown), which is likely to be the consequence of the year-long multifactorial treatment aimed at CV risk reduction. For example, in a recent study of 305 type 2 diabetic patients (mean age 58.6 years and 54.4% males) referred to a diabetes clinic for the first time with less than 5 years of diabetes duration and without CVD, CIMT > 1.0 mm was observed in 35% of subjects and the average CIMT was 0.82 mm [6]. The latter study also included a non-diabetic reference group with an average CIMT comparable to our patients (0.72 mm vs. 0.73 mm). Furthermore, the METEOR trial showed that CIMT increased 0.0131 mm/year in type 2 diabetic patients compared to non-diabetic subjects but in diabetic patients on statin therapy CIMT regressed 0.0014 mm/year [29]. In addition 10 trials including 3.443 nondiabetic and diabetic individuals have shown that statin therapy significantly reduced the progression of carotid atherosclerosis [30]. In a large cross-sectional observational population study, the average CIMT among subjects without risk factors was 0.712 mm in men and 0.682 mm in women, while in subjects with 1 CV risk factor and mean age of 61 years, CIMT was 0.765 mm [31]. Our subjects were on average 59 years of age with diabetes duration of 13 years and all had albuminuria in addition to several other CV risk factors. With longer diabetes duration CIMT increased although a decline was seen in those with the longest diabetes duration probably because of the selection force of the excess mortality in this group.

Our observed prevalences of coronary and peripheral artery disease were higher (34% and 20%) than previously reported (19% and 15%) in asymptomatic but less complicated diabetics [32 33]. Age was an independent determinant of coronary atherosclerosis, and as also found in our study, diabetes duration is a known determinant of peripheral artery disease [34]. Our patients with peripheral atherosclerosis had independently increased risk of coronary atherosclerosis and vice versa. Autopsy studies in the general population have demonstrated a close relationship between the presence of atherosclerosis in the three vascular territories[35 36] but as in accordance with a recent study, a high percentage of our patients (56/85 = 66%, Figure 1) with coronary, carotid and/or peripheral atherosclerosis demonstrated exclusively one diseased vascular territory [6]. Therefore these relations were not strong enough to examine only one artery territory and then identify all cases with atherosclerosis in the different territories.

### Vascular dysfunction

Peripheral pulse pressure wave augmentation and carotid compliance were not associated with NT-proBNP or CCS, CIMT and PAD. Noteworthy, we found that a low RHI determined by peripheral artery tonometry was an independent predictor of coronary atherosclerosis, and the RHI has been shown to be positively correlated with the endothelium-dependent coronary flow reserve [18]. In the latter study, a RHI cut-off value of < 1.35, i.e., comparable RHI ≤1.43 used in the current study, was particularly useful for determination of patients with coronary microvascular endothelial dysfunction [18]. In the Framingham offspring cohort, it was also recently shown that the RHI was associated with conventional and metabolic CV risk factors [17]. Our study confirms and extends these findings and provides evidence of a potential usability of a normal RHI for selection of asymptomatic diabetes patients at low risk of coronary atherosclerosis. Although coronary atherosclerosis as defined by CCS≥400 is a strong and independent CAD predictor, the examination is costly and associated with radiation exposure. Furthermore, only one third of our patients had coronary atherosclerosis defined by CCS≥400, and our results suggest that the RHI may contribute significantly to coronary atherosclerosis screening in these patients.

### Clinical implications

Microalbuminuria is associated with micro- and macroangiopathy and its presence is a well-established CV risk marker in type 2 diabetic patients [37]. Furthermore, previous studies suggest that NT-proBNP identifies a subgroup of patients with increased CV risk, independent of levels of microalbuminuria [4]. As discussed above, our present study did not demonstrate that NT-proBNP could be used as an independent biomarker in the detection of subclinical atherosclerosis. We did, however, discuss the potential use in the detection of subclinical left ventricular dysfunction based on other recent studies. In contrast, we suggested normal RHI for selection of patients at low risk of coronary atherosclerosis. Finally, our finding that more than half of our asymptomatic patients had signs of atherosclerosis despite intensive multifactorial treatment, is interesting. However, although coronary, carotid and peripheral atherosclerosis are known to be strong predictors of CV mortality in diabetic patients [38 39 40], the true clinical impact of the presence of atherosclerosis in different vascular territories in our present cohort of aggressively treated patients will be examined by planned prospective follow-up.

### Strengths and limitations

Our cohort is relatively selected, but this patient group is expected to have excess mortality compared to the general population. In five, seven and 17 patients CCS, peripheral systolic blood pressure and CIMT were not measured respectively, but we only included patients with available structural tests in the respective territories. We used a definition of atherosclerosis that was dependent on well-established and reasonably accurate non-invasive investigative modalities [14 16 41]. Specifically, CCS is a continuous variable reflecting the coronary artery atherosclerotic burden. Coronary atherosclerosis is, therefore, not unequivocally defined by CCS ≥400 but evidence suggests that this cut-off value defines clinical significant disease in diabetic patients with proteinuria [13]. CIMT was measured bilaterally as recently recommended [14]. Also, since determination of peripheral artery disease by ABI < 90% may not be sufficiently accurate in diabetic patients due to increased risk of peripheral arterial calcification leading to non-compressible arteries and nonvalid ABI measurements, TBI was also measured in all our patients [16]. RHI measured by peripheral artery tonometry is a well-established marker of peripheral microvascular endothelial function, that is associated with brachial and coronary artery endothelial function [18].

## Conclusion

Half of our asymptomatic patients with type 2 diabetes, albuminuria and normal P-creatinine, had atherosclerosis in one or more vascular territories despite receiving intensive multifactorial treatment aimed at CV risk reduction. In this cohort, patients with elevated P-NT-proBNP did not have more subclinical atherosclerosis, and although P-NT-proBNP was increased in patients with multiple atherosclerotic lesions it was not an independent CV risk factor. Reactive hyperaemia index determined by peripheral arterial tonometry showed potential for coronary atherosclerosis screening.

## List of abbreviations

ABI: Ankle-brachial index; AI: Augmentation index; AUC: Areas under the ROC curve; BMI: Body mass index; CAG: Coronary angiography; CCS: Coronary calcium score; CI: Confidence interval; CIMT: Carotid intima-media thickness; CTA: CT angiography; CV: Cardiovascular; MPI: Myocardial perfusion imaging; NT-proBNP: N-terminal pro brain natriuretic peptide; OR: Odds ratio; PAD: Peripheral artery disease; RAAS: Renin-angiotensin-aldosterone-system; RHI: Reactive hyperaemia index; ROC: Receiver operating characteristic; SD: Standard deviation; TBI: Toe-brachial index; WHO: World Health Organisation.

## Competing interests

Dr. Rossing reports having received lecture fees from Novartis and Boehringer Ingelheim, and research grant from Novartis, has served as a consultant for Merck, and having equity interest in NovoNordisk. Dr. Parving reports having served as a consultant for Novartis, Merck, Pfizer and Sanofi-Aventis, having equity interest in Merck and NovoNordisk and having received lecture fees from Novartis, Merck, Pfizer and Sanofi-Aventis. Dr. Parving has received grant support from Novartis, AstraZeneca and Sanofi-Aventis.

## Authors' contributions

HR: researched data, contributed to discussion, wrote manuscript

PKJ, PR, PRH, HHP, NW, AK, CLP, KW: researched data, contributed to discussion, reviewed/edited manuscript

All authors read and approved the final manuscript.

## References

[B1] Gaede P Lund-Andersen H Parving HH Effect of a multifactorial intervention on mortality in type 2 diabetes N Engl J Med 2008 358 6 580 91 10.1056/NEJMoa0706245 18256393

[B2] Doust JA Pietrzak E Dobson A How well does B-type natriuretic peptide predict death and cardiac events in patients with heart failure: systematic review Br Med J 2005 330 7492 625 10.1136/bmj.330.7492.625 PMC554905 15774989

[B3] Wang TJ Larson MG Levy D Plasma natriuretic peptide levels and the risk of cardiovascular events and death N Engl J Med 2004 350 7 655 63 10.1056/NEJMoa031994 14960742

[B4] Tarnow L Gall MA Hansen BV Plasma N-terminal pro-B-type natriuretic peptide and mortality in type 2 diabetes Diabetologia 2006 49 2256 62 10.1007/s00125-006-0359-4 16937127

[B5] Abdullah SM Khera A Das SR Relation of coronary atherosclerosis determined by electron beam computed tomography and plasma levels of n-terminal pro-brain natriuretic peptide in a multiethnic population-based sample (the Dallas Heart Study) Am J Cardiol 2005 96 9 1284 9 10.1016/j.amjcard.2005.06.073 16253599

[B6] Poulsen MK Henriksen JE Dahl J Myocardial ischemia, carotid, and peripheral arterial disease and their interrelationship in type 2 diabetes patients J Nucl Cardiol 2009 16 6 878 87 10.1007/s12350-009-9118-5 19685102

[B7] Christensen LL Anker-Nielsen A Almdal TP Development in Quality of Treatment in Complicated Type 2 Diabetes Assessed by the Proportion of Patients Reaching ADA Goals Diab 2010 59 A180 A181

[B8] Reinhard H Hansen PR Persson F Elevated NT-proBNP and coronary calcium score in relation to coronary artery disease in asymptomatic type 2 diabetic patients with elevated urinary albumin excretion rate Nephrol Dial Transplant 2011 10.1093/ndt/gfr009 21372253

[B9] Agatston AS Janowitz WR Hildner FJ Quantification of coronary artery calcium using ultrafast computed tomography J Am Coll Cardiol 1990 15 4 827 32 10.1016/0735-1097(90)90282-T 2407762

[B10] Simons DB Schwartz RS Edwards WD Noninvasive definition of anatomic coronary artery disease by ultrafast computed tomographic scanning: a quantitative pathologic comparison study J Am Coll Cardiol 1992 20 5 1118 26 10.1016/0735-1097(92)90367-V 1401612

[B11] Rumberger JA Simons DB Fitzpatrick LA Coronary artery calcium area by electron-beam computed tomography and coronary atherosclerotic plaque area. A histopathologic correlative study Circulation 1995 92 8 2157 62 7554196 10.1161/01.cir.92.8.2157

[B12] Oudkerk M Stillman AE Halliburton SS Coronary artery calcium screening: current status and recommendations from the European Society of Cardiac Radiology and North American Society for Cardiovascular Imaging Int J Cardiovasc Imaging 2008 24 6 645 71 10.1007/s10554-008-9319-z 18504647 PMC2493606

[B13] Chiu YW Adler SG Budoff MJ Coronary artery calcification and mortality in diabetic patients with proteinuria Kidney Int 2010 77 12 1107 14 10.1038/ki.2010.70 20237457

[B14] Laurent S Cockcroft J Van BL Expert consensus document on arterial stiffness: methodological issues and clinical applications Eur Heart J 2006 27 21 2588 605 10.1093/eurheartj/ehl254 17000623

[B15] Lassen NA Tvedegaard E Jeppesen FI Distal blood pressure measurement in occlusive arterial disease, strain gauge compared to xenon-133 Angiology 1972 23 4 211 7 10.1177/000331977202300405 5030556

[B16] Carter SA Lezack JD Digital systolic pressures in the lower limb in arterial disease Circulation 1971 43 905 14 5578864 10.1161/01.cir.43.6.905

[B17] Hamburg NM Keyes MJ Larson MG Cross-sectional relations of digital vascular function to cardiovascular risk factors in the Framingham Heart Study Circulation 2008 117 19 2467 74 10.1161/CIRCULATIONAHA.107.748574 18458169 PMC2734141

[B18] Bonetti PO Pumper GM Higano ST Noninvasive identification of patients with early coronary atherosclerosis by assessment of digital reactive hyperemia Journal of the American College of Cardiology 2004 44 11 2137 41 10.1016/j.jacc.2004.08.062 15582310

[B19] Higashi Y Sasaki S Nakagawa K A noninvasive measurement of reactive hyperemia that can be used to assess resistance artery endothelial function in humans Am J Cardiol 2001 87 1 121 5 A9. 10.1016/S0002-9149(00)01288-1 11137850

[B20] Hamilton PK Lockhart CJ Quinn CE Arterial stiffness: clinical relevance, measurement and treatment Clin Sci (Lond) 2007 113 4 157 70 10.1042/CS20070080 17623012

[B21] Heffernan KS Patvardhan EA Hession M Elevated augmentation index derived from peripheral arterial tonometry is associated with abnormal ventricular-vascular coupling Clin Physiol Funct Imaging 2010 30 5 313 7 20545714 10.1111/j.1475-097X.2010.00943.x PMC3148895

[B22] Reinhard H Hansen PR Persson F Elevated NT-proBNP and coronary calcium score in relation to coronary artery disease in asymptomatic type 2 diabetic patients with elevated urinary albumin excretion rate Nephrol Dial Transplant 2011 10.1093/ndt/gfr009 21372253

[B23] Gaede P Hildebrandt P Hess G Plasma N-terminal pro-brain natriuretic peptide as a major risk marker for cardiovascular disease in patients with type 2 diabetes and microalbuminuria Diabetologia 2005 48 1 156 63 10.1007/s00125-004-1607-0 15619076

[B24] Magnusson M Jovinge S Shahgaldi K Brain natriuretic peptide is related to diastolic dysfunction whereas urinary albumin excretion rate is related to left ventricular mass in asymptomatic type 2 diabetes patients Cardiovasc Diabetol 2010 9 2 10.1186/1475-2840-9-2 20078898 PMC2817679

[B25] Romano S Di MM Fratini S Early diagnosis of left ventricular diastolic dysfunction in diabetic patients: a possible role for natriuretic peptides Cardiovasc Diabetol 2010 9 89 10.1186/1475-2840-9-89 21162718 PMC3019186

[B26] Mangge H Almer G Zelzer S N-terminal pro-B-type natriuretic peptide in early and advanced phases of obesity Clin Chem Lab Med 2011 10.1515/CCLM.2011.627 21663466

[B27] Novo G Corrado E Tortorici E Cardiac risk stratification in elective non-cardiac surgery: role of NT-proBNP Int Angiol 2011 30 3 242 6 21617607

[B28] Macheret F Boerrigter G McKie P Pro-B-type natriuretic peptide(1-108) circulates in the general community: plasma determinants and detection of left ventricular dysfunction J Am Coll Cardiol 2011 57 12 1386 95 10.1016/j.jacc.2011.01.005 21414536 PMC3927966

[B29] Crouse JR III Raichlen JS Riley WA Effect of rosuvastatin on progression of carotid intima-media thickness in low-risk individuals with subclinical atherosclerosis: the METEOR Trial JAMA 2007 297 12 1344 53 10.1001/jama.297.12.1344 17384434

[B30] Kang S Wu Y Li X Effects of statin therapy on the progression of carotid atherosclerosis: a systematic review and meta-analysis Atherosclerosis 2004 177 2 433 42 10.1016/j.atherosclerosis.2004.08.005 15530920

[B31] Touboul PJ Labreuche J Vicaut E Country-based reference values and impact of cardiovascular risk factors on carotid intima-media thickness in a French population: the 'Paroi Arterielle et Risque Cardio-Vasculaire' (PARC) Study Cerebrovasc Dis 2009 27 4 361 7 10.1159/000202013 19218802

[B32] Scholte AJ Schuijf JD Kharagjitsingh AV Prevalence of coronary artery disease and plaque morphology assessed by multi-slice computed tomography coronary angiography and calcium scoring in asymptomatic patients with type 2 diabetes Heart 2008 94 3 290 5 10.1136/hrt.2007.121921 17646190

[B33] Faglia E Caravaggi C Marchetti R Screening for peripheral arterial disease by means of the ankle-brachial index in newly diagnosed Type 2 diabetic patients Diabet Med 2005 22 10 1310 4 10.1111/j.1464-5491.2005.01612.x 16176188

[B34] Norgren L Hiatt WR Dormandy JA Inter-Society Consensus for the Management of Peripheral Arterial Disease (TASC II) Eur J Vasc Endovasc Surg 2007 33 Suppl 1 S1 75 17140820 10.1016/j.ejvs.2006.09.024

[B35] Dalager S Paaske WP Kristensen IB Artery-related differences in atherosclerosis expression: implications for atherogenesis and dynamics in intima-media thickness Stroke 2007 38 10 2698 705 10.1161/STROKEAHA.107.486480 17761918

[B36] Dalager S Falk E Kristensen IB Plaque in superficial femoral arteries indicates generalized atherosclerosis and vulnerability to coronary death: an autopsy study J Vasc Surg 2008 47 2 296 302 10.1016/j.jvs.2007.10.037 18241752

[B37] Ninomiya T Perkovic V de Galan BE Albuminuria and kidney function independently predict cardiovascular and renal outcomes in diabetes J Am Soc Nephrol 2009 20 8 1813 21 10.1681/ASN.2008121270 19443635 PMC2723977

[B38] Raggi P Shaw LJ Berman DS Prognostic value of coronary artery calcium screening in subjects with and without diabetes J Am Coll Cardiol 2004 43 9 1663 9 10.1016/j.jacc.2003.09.068 15120828

[B39] Bernard S Serusclat A Targe F Incremental predictive value of carotid ultrasonography in the assessment of coronary risk in a cohort of asymptomatic type 2 diabetic subjects Diabetes Care 2005 28 5 1158 62 10.2337/diacare.28.5.1158 15855582

[B40] Norman PE Davis WA Bruce DG Peripheral arterial disease and risk of cardiac death in type 2 diabetes: the Fremantle Diabetes Study Diabetes Care 2006 29 3 575 80 10.2337/diacare.29.03.06.dc05-1567 16505509

[B41] Budoff MJ Gul KM Expert review on coronary calcium Vasc Health Risk Manag 2008 4 2 315 24 18561507 10.2147/vhrm.s1160 PMC2496978

